# A Micromechanical Fatigue Limit Stress Model of Fiber-Reinforced Ceramic-Matrix Composites under Stochastic Overloading Stress

**DOI:** 10.3390/ma13153304

**Published:** 2020-07-24

**Authors:** Longbiao Li

**Affiliations:** College of Civil Aviation, Nanjing University of Aeronautics and Astronautics, No. 29 Yudao St., Nanjing 210016, China; llb451@nuaa.edu.cn

**Keywords:** fatigue limit stress, ceramic-matrix composites (CMCs), stochastic overloading stress, fiber failure

## Abstract

Fatigue limit stress is a key design parameter for the structure fatigue design of composite materials. In this paper, a micromechanical fatigue limit stress model of fiber-reinforced ceramic-matrix composites (CMCs) subjected to stochastic overloading stress is developed. The fatigue limit stress of different carbon fiber-reinforced silicon carbide (C/SiC) composites (i.e., unidirectional (UD), cross-ply (CP), 2D, 2.5D, and 3D C/SiC) is predicted based on the micromechanical fatigue damage models and fatigue failure criterion. Under cyclic fatigue loading, the fatigue damage and fracture under stochastic overloading stress at different applied cycle numbers are characterized using two parameters of fatigue life decreasing rate and broken fiber fraction. The relationships between the fatigue life decreasing rate, stochastic overloading stress level and corresponding occurrence applied cycle number, and broken fiber fraction are analyzed. Under the same stochastic overloading stress level, the fatigue life decreasing rate increases with the occurrence applied cycle of stochastic overloading, and thus, is the highest for the cross-ply C/SiC composite and lowest for the 2.5D C/SiC composite. Among the UD, 2D, and 3D C/SiC composites, at the initial stage of cyclic fatigue loading, under the same stochastic overloading stress, the fatigue life decreasing rate of the 3D C/SiC is the highest; however, with the increasing applied cycle number, the fatigue life decreasing rate of the UD C/SiC composite is the highest. The broken fiber fraction increases when stochastic overloading stress occurs, and the difference of the broken fiber fraction between the fatigue limit stress and stochastic overloading stress level increases with the occurrence applied cycle.

## 1. Introduction

Ceramic-matrix composites (CMCs) possess high specific strength and specific modulus, high temperature resistance, and have already been applied on hot section components of commercial aero engines [1,2,3]. To ensure the reliability and safety of CMC components, it is necessary to develop performance evaluation, damage evolution, strength, and life prediction tools for airworthiness certification [4].

Under cyclic fatigue loading, matrix cracking, interface debonding, interface wear, and fiber fracture occur with the applied cycle, and these fatigue damage mechanisms degrade the mechanical performance of fiber-reinforced CMCs [5,6,7]. Fatigue limit stress is a key parameter for the design of CMC components. However, fatigue limit stress of CMCs depends on many factors, i.e., fiber characteristic and fiber properties [8,9], loading frequency [10,11], temperature [12,13], and testing conditions [14,15,16]. Under cyclic fatigue loading, stochastic overloading stress may occur due to a special operation condition of the aero engine, which can affect the internal fatigue damage evolution and lifetime of CMCs [17,18]. Reynaud [5], Evans [19], and Li [20,21,22] developed micromechanical fatigue life prediction methods for fiber-reinforced CMCs considering different fatigue damage mechanisms. The degradation rate of the fiber/matrix interface shear stress and fiber strength affects the fatigue life and fatigue limit stress. However, in the developed micromechanical model, the effect of stochastic overloading stress on fatigue limit stress has not been considered.

In this paper, a micromechanical fatigue limit stress model of fiber-reinforced CMCs subjected to stochastic overloading stress is developed. The fatigue limit stress for different carbon fiber-reinforced silicon carbide (C/SiC) composites is predicted based on the micromechanical fatigue damage models and fatigue failure criterion. The relationships between the fatigue life decreasing rate, stochastic overloading stress level and corresponding occurrence applied cycle number, and broken fiber fraction are analyzed.

## 2. Theoretical Model

**Figure 1:**
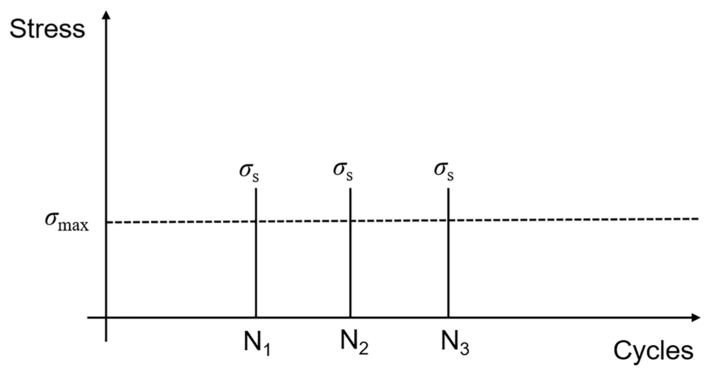
Diagram of stochastic overloading stress under cyclic fatigue loading.

When stochastic overloading stress occurs under cyclic fatigue loading, the fatigue damage evolution of matrix cracking, interface debonding, and fiber failure are affected. Figure 1 shows stochastic overloading stress *σ*_s_ occurred at different applied cycle numbers. In the present analysis, the overloading stress *σ*_s_ remains the same at applied cycle numbers *N*_1_, *N*_2_, and *N*_3_.

Based on the global load sharing (GLS) criterion, under stochastic overloading stress, the stress carried by intact and broken fiber is determined by Equation (1) [23].

(1) $$\frac{\sigma_{s}}{V_{f}}=\Phi_{s}\left(1-P_{f}\right)+\Phi_{b}P_{f}$$

where *V*_f_ is the fiber volume, Φ_s_ is the intact fiber stress under stochastic overloading stress, Φ_b_ is the stress carried by broken fiber, and *P*_f_ is the fiber failure probability, and can be determined by Equation (2).

(2) $$P_{f}=1-\exp\left[-\Theta \Omega^{m}{\left(\frac{\Phi_{s}}{\sigma_{c}}\right)}^{m+1}\right]$$

(3) $$\Phi_{b}=\frac{\Phi_{s}}{\Theta \Omega^{m}P_{f}}{\left(\frac{\sigma_{c}}{\Phi_{s}}\right)}^{m+1}\left\{1-\exp\left[-\Theta \Omega^{m}{\left(\frac{\Phi_{s}}{\sigma_{c}}\right)}^{m+1}\right]\right\}-\frac{\Phi_{s}}{P_{f}}\exp\left[-\Theta \Omega^{m}{\left(\frac{\Phi_{s}}{\sigma_{c}}\right)}^{m+1}\right]$$

where *m* is the fiber Weibull modulus, and *σ*_c_ is the fiber characteristic strength, and Θ and Ω denote the degradation rate of the interface shear stress and fiber strength and can be determined by Equations (4) and (5), respectively.

(4) $$\Theta \left(N\right)=\frac{1}{1-\left(1-\varphi \right)\left[1-\exp\left(-\omega N^{\lambda }\right)\right]}$$

(5) $$\Omega \left(N\right)=\frac{1}{1-p_{1}{\left(\log N\right)}^{p_{2}}}$$

where *φ* is the ratio between the steady interface shear stress and initial interface shear stress, *ω* and *λ* are the interface wear model parameter, and *p*_1_ and *p*_2_ are the fiber strength degradation model parameter.

Substituting Equations (2) and (3) into Equation (1), the relation between the applied stress and fiber intact stress is determined by Equations (6).

(6) $$\frac{\sigma_{s}}{V_{f}}=\frac{\Phi_{s}}{\Theta \Omega^{m}}{\left(\frac{\sigma_{c}}{\Phi_{s}}\right)}^{m+1}\left\{1-\exp\left[-\Theta \Omega^{m}{\left(\frac{\Phi_{s}}{\sigma_{c}}\right)}^{m+1}\right]\right\}$$

Using Equations (4)–(6), the intact fiber stress under stochastic overloading stress can be obtained with the occurrence applied cycle number. Substituting the intact fiber stress under stochastic overloading stress into Equation (2), the fraction of broken fiber under stochastic overloading stress can be obtained. Under cyclic fatigue loading, when the fiber failure probability approaches the critical value, the composite fatigue fractures. The fatigue limit stress of CMCs at room temperature can be obtained using the developed life prediction model and fatigue limit cycle number *N*_limit_.

The fatigue life decreasing rate is defined by Equation (7).

(7) $$\Lambda =\frac{N_{f}\left(\sigma_{\mathrm{limit}}\right)-N_{f}\left(\sigma_{s}\right)}{N_{f}\left(\sigma_{\mathrm{limit}}\right)}$$

where *N*_f_(*σ*_limit_) is the fatigue failure cycle number under fatigue limit stress, and *N*_f_(*σ*_s_) is the fatigue failure cycle number under stochastic overloading stress.

## 3. Experimental Comparisons

Under cyclic fatigue loading, stochastic overloading stress affects the fatigue damage evolution, i.e., increasing the fiber failure probability, and decreasing the fatigue life. In this section, the fatigue limit stress of different C/SiC composites is predicted. The material properties and fatigue damage model parameters of the C/SiC composite are listed in Table 1. Under fatigue limit stress, stochastic overloading occurring in different applied cycle numbers can decrease the fatigue life. Using the developed fiber failure model in Equation (2) and the fatigue damage models in Equations (4) and (5), the effect of the stochastic overloading stress level and corresponding occurrence cycle number on the fatigue limit stress and corresponding fatigue life is analyzed. The relationships between the stochastic overloading stress level, occurrence cycle number, broken fiber fraction, and fatigue limit stress are established.

### 3.1. Unidirectional C/SiC Composite

The unidirectional T−700^TM^ carbon fiber-reinforced silicon carbide composite was fabricated using the hot-pressing (HP) method. Low pressure chemical vapor infiltration was employed to deposit approximately 5−20 layers of PyC/SiC with the mean thickness of 0.2 μm. The nano-SiC powder and sintering additives were ball milled for 4 h using SiC balls. After drying, the powders were dispersed in xylene with polycarbonsilane (PCS) to form the slurry. Carbon fiber tows were infiltrated by the slurry and wound to form aligned unidirectional composite sheets. After drying, the sheets were cut to a size of 150 mm × 150 mm and pyrolyzed in argon. Then the sheets were stacked in a graphite die and sintered by hot pressing. The dog-bone shaped specimens were cut from 150 mm × 150 mm panels by water cutting. The tension–tension fatigue tests were conducted on a MTS Model 809 servo hydraulic load-frame (MTS Systems Corp., Minneapolis, MN, USA). The fatigue experiments were in a sinusoidal wave form with a loading frequency *f* = 10 Hz. The fatigue load ratio (*σ*_min_/*σ*_max_) was *R* = 0.1. The fatigue tests were conducted under load control at room temperature.

**Figure 2:**
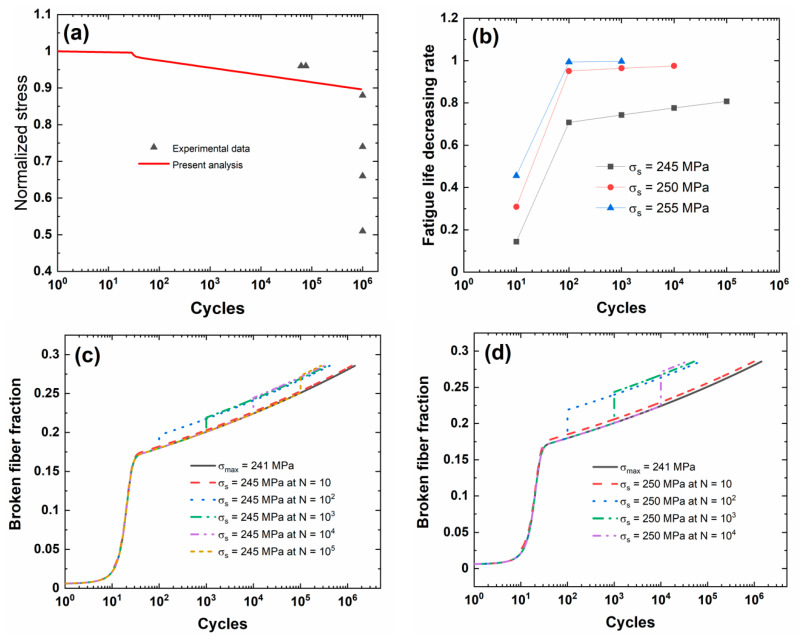

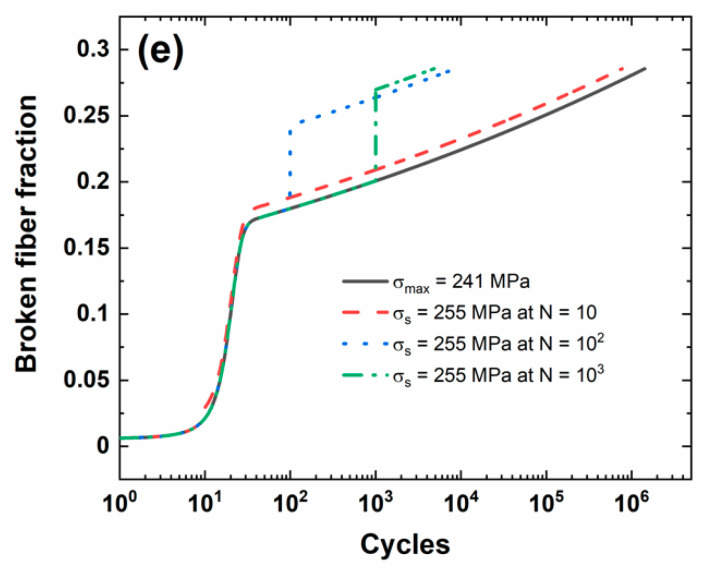
(**a**) Experimental and predicted fatigue life S−N curves; (**b**) the fatigue life decreasing rate versus occurrence applied cycle number curve for different stochastic loading stress levels; (**c**) the broken fiber fraction versus applied cycle number curves under *σ*_limit_ = 241 MPa and stochastic overloading stress *σ*_s_ = 245 MPa at *N*_s_ = 10, 10^2^, 10^3^, 10^4^, and 10^5^; (**d**) the broken fiber fraction versus applied cycle number curves under *σ*_limit_ = 241 MPa and stochastic overloading stress *σ*_s_ = 250 MPa at *N*_s_ = 10, 10^2^, 10^3^, 10^4^, and 10^5^; and, (**e**) the broken fiber fraction versus applied cycle number curves under *σ*_limit_ = 241 MPa and stochastic overloading stress *σ*_s_ = 255 MPa at *N*_s_ = 10, 10^2^, and 10^3^ of unidirectional C/SiC composite.

Figure 2 shows the experimental and predicted fatigue life S−N curves of the unidirectional C/SiC composite. When the fatigue limit applied cycle number is defined to be *N*_limit_ = 10^6^, the corresponding predicted fatigue limit stress is approximately *σ*_limit_ = 241 MPa (approximately 89.2%*σ*_uts_).

Figure 2b shows the fatigue life decreasing rate versus the occurrence cycle number of stochastic overloading curves for different stochastic overloading stress levels of *σ*_s_ = 245, 250, and 255 MPa (i.e., approximately 1.016, 1.037, 1.058 of fatigue limit stress). During the application of CMC components, the overloading stress level is not high, and the low overloading stress level is chosen for analysis. Under the same stochastic overloading stress level of *σ*_s_ = 245, 250, and 255 MPa, the fatigue life decreasing rate increases with the occurrence applied cycle of stochastic overloading (i.e., *N*_s_ = 10, 10^2^, 10^3^, 10^4^, and 10^5^). Under *σ*_s_ = 245 MPa, the fatigue life decreasing rate increases from Λ = 0.14392 at *N*_s_ = 10 to Λ = 0.8074 at *N*_s_ = 10^5^; under *σ*_s_ = 250 MPa, the fatigue life decreasing rate increases from Λ = 0.3091 at *N*_s_ = 10 to Λ = 0.97479 at *N*_s_ = 10^4^; and under *σ*_s_ = 255 MPa, the fatigue life decreasing rate increases from Λ = 0.4559 at *N*_s_ = 10 to Λ = 0.9966 at *N*_s_ = 10^3^. When the applied cycle is between *N*_s_ = 10 and 10^2^, the fatigue life decreasing rate increases rapidly with the occurrence applied cycle; however, when the applied cycle is higher than *N*_s_ = 10^2^, the fatigue life decreasing rate increases slowly with the applied cycle. At the initial stage of cyclic fatigue loading, the fatigue damage mechanisms of matrix cracking, interface debonding and wear depend on the fatigue peak stress level. The occurrence of stochastic overloading stress at the initial stage of cyclic fatigue loading deteriorates fatigue damage evolution, i.e., decreasing matrix crack spacing, increasing interface debonding length and broken fiber fraction; however, when matrix cracking and interface wear approach a steady-state, the effect of stochastic overloading stress on fatigue damage or fatigue life decreasing rate decreases.

Figure 2c–e shows the broken fiber fraction versus the applied cycle number curves for different stochastic overloading stress levels and occurrence applied cycle numbers. The broken fiber fraction increases when stochastic overloading stress occurs, and the difference of the broken fiber fraction between original peak stress and stochastic overloading stress level increases with the applied cycle number.

Table 2 shows the fatigue limit stress and broken fiber fraction at different occurrence cycle numbers and stochastic overloading stress. When stochastic overloading stress *σ*_s_ = 245 MPa occurs at applied cycles *N*_s_ = 10, 10^2^, 10^3^, 10^4^, and 10^5^, the broken fiber fraction increases from *P*_f_ = 0.02113, 0.17991, 0.20077, 0.22431, and 0.25085 under *σ*_limit_ = 241 MPa to *P*_f_ = 0.02329, 0.19662, 0.21915, 0.2445, and 0.27298; when stochastic overloading stress *σ*_s_ = 250 MPa occurs at applied cycles *N*_s_ = 10, 10^2^, 10^3^, and 10^4^, the broken fiber fraction increases to *P*_f_ = 0.02626, 0.21897, 0.24365, and 0.27131; finally, when stochastic overloading stress *σ*_s_ = 255 MPa occurs at applied cycles *N* = 10, 10^2^, and 10^3^, the broken fiber fraction increases to *P*_f_ = 0.02952, 0.24295, and 0.26984.

### 3.2. Cross-Ply C/SiC Composite

The cross-ply T−700^TM^ carbon fiber-reinforced silicon carbide composite was fabricated using the hot-pressing (HP) method, which offered the ability to fabricate dense composites via a liquid phase sintering method at a low temperature. The fiber volume is *V*_f_ = 0.4, and the average tensile strength is approximately *σ*_uts_ = 124 MPa. The dog-bone shaped specimens were cut from 150 mm × 150 mm panels by water cutting. The tension–tension fatigue tests were conducted on an MTS Model 809 servo hydraulic load-frame (MTS Systems Corp., Minneapolis, MN, USA). The fatigue experiments were in a sinusoidal wave form with a loading frequency *f* = 10 Hz. The fatigue load ratio (*σ*_min_/*σ*_max_) was *R* = 0.1. The fatigue tests were conducted under load control at room temperature.

**Figure 3:**
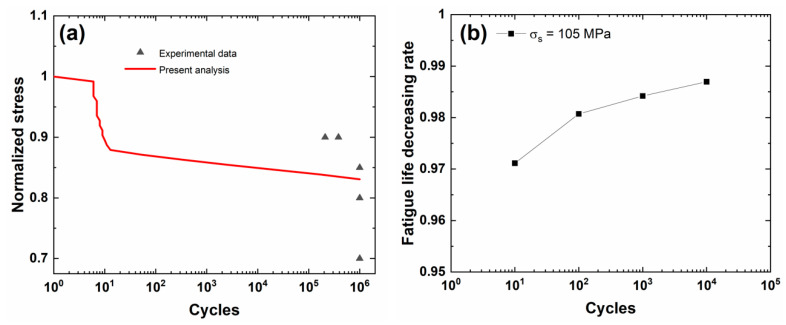

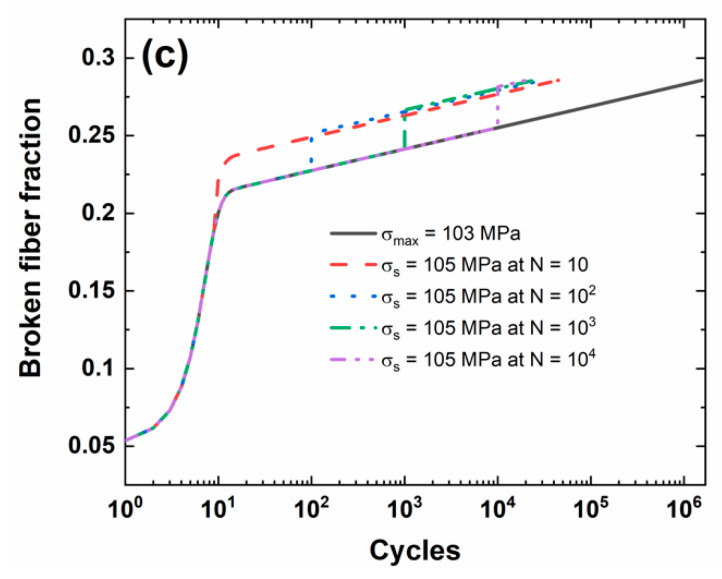
(**a**) Experimental and predicted fatigue life S−N curves; (**b**) the fatigue life decreasing rate versus occurrence applied cycle curve; and, (**c**) the broken fiber fraction versus applied cycle curves under *σ*_limit_ = 103 MPa and stochastic overloading stress *σ*_s_ = 105 MPa at *N*_s_ = 10, 10^2^, 10^3^, and 10^4^ of cross-ply C/SiC composite.

Figure 3a shows experimental and predicted fatigue life S−N curves of the cross-ply C/SiC composite. When the fatigue limit applied cycle number is defined to be *N*_limit_ = 10^6^, the corresponding predicted fatigue limit stress is approximately *σ*_limit_ = 103 MPa (approximately 83%*σ*_uts_).

Figure 3b shows the fatigue life decreasing rate versus the occurrence applied cycle number of stochastic overloading stress curves under *σ*_s_ = 105 MPa (approximately 1.019 fatigue limit stress). During the application of CMC components, the overloading stress level is not high, and the low overloading stress level is chosen for analysis. The fatigue life decreasing rate increases with the occurrence applied cycle number of stochastic overloading. Under *σ*_s_ = 105 MPa, the fatigue life decreasing rate increases from Λ = 0.97114 at *N*_s_ = 10 to Λ = 0.98696 at *N*_s_ = 10^4^. When the occurrence applied cycle number is between *N*_s_ = 10 and 10^2^, the fatigue life decreasing rate increases rapidly; however, when the occurrence applied cycle is between *N*_s_ = 10^2^ and 10^4^, the fatigue life decreasing rate increases slowly. The occurrence of stochastic overloading stress at the initial stage of cyclic fatigue loading deteriorates the fatigue damage evolution, i.e., decreasing matrix crack spacing in transverse and longitudinal plies, increasing interface debonding length, and broken fiber fraction; however, when matrix cracking and interface wear approach a steady-state, the effect of stochastic overloading on the fatigue damage or fatigue life decreasing rate decreases.

Figure 3c shows the broken fiber fraction versus applied cycle number curves for different occurrence cycle numbers (i.e., *N*_s_ = 10, 10^2^, 10^3^, and 10^4^). The broken fiber fraction increases when stochastic overloading stress occurs, and the difference of the broken fiber fraction between the original peak stress and stochastic overloading stress level increases with the applied cycle.

Table 3 shows the fatigue limit stress and broken fiber fraction under a stochastic overloading stress of *σ*_s_ = 105 MPa at different occurrence applied cycle numbers. When stochastic overloading stress *σ*_s_ = 105 MPa occurs at applied cycle numbers of *N*_s_ = 10, 10^2^, 10^3^, and 10^4^, the broken fiber fraction increases from *P*_f_ = 0.20047, 0.22748, 0.24133, and 0.25508 under *σ*_limit_ = 103 MPa to *P*_f_ = 0.22205, 0.25148, 0.26653, and 0.28143.

### 3.3. The 2D C/SiC Composite

The 2D T−300^TM^ carbon fiber-reinforced silicon carbide composite was fabricated using the chemical vapor infiltration (CVI) method. It contained 26 plies of plain-weave cloth in a (0°/90°) lay-up. Fiber preform was given a pyrolytic carbon coating. The fiber volume was 45%, and density was 1.93–1.98 g/cm^3^, and the porosity was approximately 22%. The dog-bone shaped specimens were cut from 200 mm × 200 mm panels using diamond tooling. The tension–tension fatigue tests at room temperature were conducted on a servohydraulic load-frame that was equipped with edge-loaded grips. The fatigue experiments were performed under load control at a sinusoidal wave form and a loading frequency *f* = 10 Hz. The fatigue load ratio (*σ*_min_/*σ*_max_) was *R* = 0.1. The average tensile strength was approximately *σ*_uts_ = 420 MPa.

**Figure 4:**
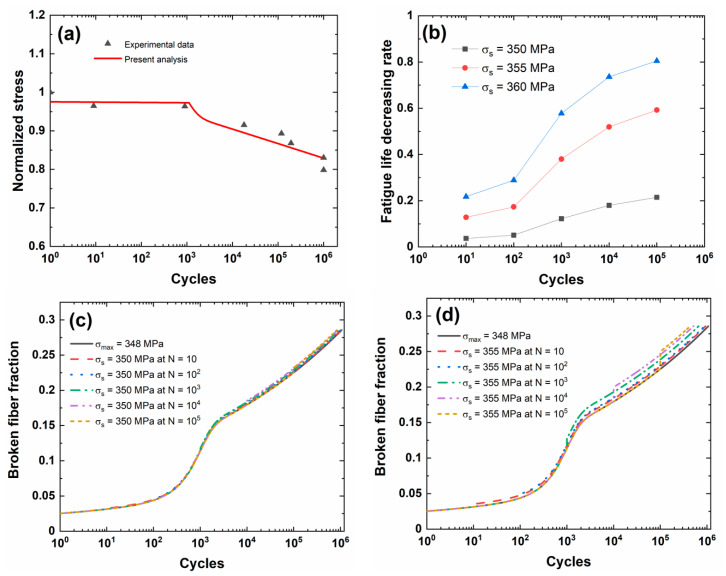

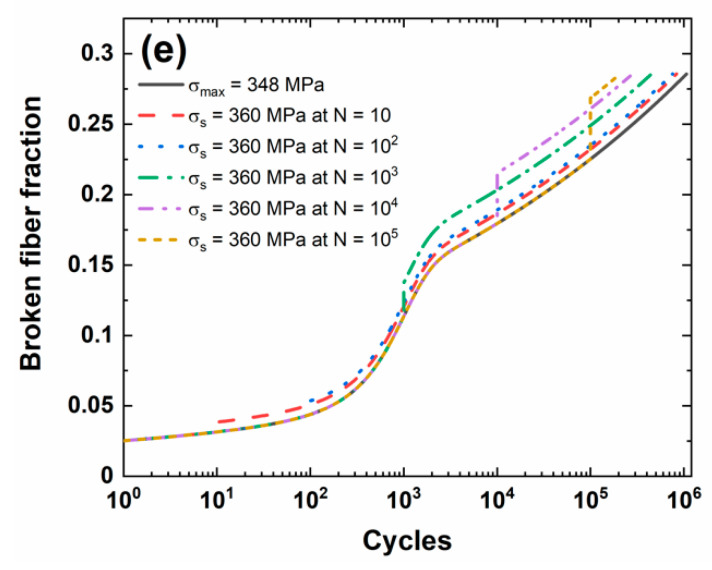
(**a**) Experimental and predicted fatigue life S−N curves; (**b**) the fatigue life decreasing rate versus occurrence applied cycle curve for different stochastic overloading stress levels; (**c**) the broken fiber fraction versus applied cycle curves under *σ*_limit_ = 348 MPa and stochastic overloading stress *σ*_s_ = 350 MPa at *N*_s_ = 10, 10^2^, 10^3^, 10^4^, and 10^5^; (**d**) the broken fiber fraction versus applied cycle curves under *σ*_limit_ = 348 MPa and stochastic overloading stress *σ*_s_ = 355 MPa at *N*_s_ = 10, 10^2^, 10^3^, 10^4^, and 10^5^; and, (**e**) the broken fiber fraction versus applied cycle curves under *σ*_limit_ = 348 MPa and stochastic overloading stress *σ*_s_ = 360 MPa at *N*_s_ = 10, 10^2^, 10^3^, 10^4^, and 10^5^ of 2D C/SiC composite.

Figure 4a shows experimental and predicted fatigue life S−N curves of the 2D C/SiC composite. When the fatigue limit applied cycle number is defined to be *N*_limit_ = 10^6^, the corresponding predicted fatigue limit stress is approximately *σ*_limit_ = 348 MPa (approximately 82.8%*σ*_uts_).

Figure 4b shows the fatigue life decreasing rate versus the occurrence applied cycle number of stochastic overloading stress curves for different stochastic overloading stress levels of *σ*_s_ = 350, 355, and 360 MPa (i.e., approximately 1.005, 1.02, 1.034 fatigue limit stress). During the application of CMC components, the overloading stress level is not high, and the low overloading stress level is chosen for analysis. Under the same stochastic overloading stress level, the fatigue life decreasing rate increases with the occurrence applied cycle of stochastic overloading. Under *σ*_s_ = 350 MPa, the fatigue life decreasing rate increases from Λ = 0.03699 at *N*_s_ = 10 to Λ = 0.21481 at *N*_s_ = 10^5^; under *σ*_s_ = 355 MPa, the fatigue life decreasing rate increases from Λ = 0.12826 at *N*_s_ = 10 to Λ = 0.59226 at *N*_s_ = 10^5^; under *σ*_s_ = 360 MPa, the fatigue life decreasing rate increases from Λ = 0.21723 at *N*_s_ = 10 to Λ = 0.80517 at *N*_s_ = 10^5^. When the occurrence applied cycle is between *N*_s_ = 10 and 10^2^, the fatigue life decreasing rate increases slowly with the occurrence applied cycle; however, when the occurrence applied cycle is between *N*_s_ = 10^2^ and 10^5^, the fatigue life decreasing rate increases rapidly with the applied cycle. For 2D C/SiC, the fatigue damage is not sensitive to stochastic overloading stress at the initial stage of cyclic fatigue loading; however, with the applied cycles increasing, the fatigue damage extent increases, leading to the rapid increase in the fatigue life decreasing rate with the occurrence cycle number of the stochastic overloading stress.

Figure 4c–e shows the broken fiber fraction versus the applied cycle number curves for different stochastic overloading stress levels and occurrence cycle numbers. The broken fiber fraction increases when stochastic overloading stress occurs, and the difference of the broken fiber fraction between the original peak stress and stochastic overloading stress level increases with the applied cycle.

Table 4 shows the fatigue limit stress and broken fiber fraction at different occurrence cycle numbers and stochastic overloading stress. When stochastic overloading stress *σ*_s_ = 350 MPa occurs at applied cycles *N*_s_ = 10, 10^2^, 10^3^, 10^4^, and 10^5^, the broken fiber fraction increases from *P*_f_ = 0.03153, 0.04397, 0.11358, 0.1795, and 0.22527 under *σ*_limit_ = 348 MPa to *P*_f_ = 0.03261, 0.04547, 0.11731, 0.18516, and 0.23215; when stochastic overloading stress *σ*_s_ = 355 MPa occurs at applied cycles *N*_s_ = 10, 10^2^, 10^3^, 10^4^, and 10^5^, the broken fiber fraction increases to *P*_f_ = 0.03546, 0.04941, 0.12704, 0.19983, and 0.24996; finally, when stochastic overloading stress *σ*_s_ = 360 MPa occurs at applied cycles *N* = 10, 10^2^, 10^3^, 10^4^, and 10^5^, the broken fiber fraction increases to *P*_f_ = 0.0385, 0.05361, 0.13736, 0.2153, and 0.26861.

### 3.4. The 2.5D C/SiC Composite

The 2.5D T−300^TM^ carbon fiber-reinforced silicon carbide composite was fabricated using the chemical vapor infiltration (CVI) method. Low pressure CVI was employed to deposit a pyrolytic carbon layer and a silicon matrix. A thin pyrolytic carbon layer was deposited on the surface of the carbon fiber as the interfacial layer with C_3_H_8_ at 800 °C. Methyltrichlorosilane (MTS, CH_3_ SiCl_3_) was used as a gas source for the deposition of the SiC matrix. The conditions for deposition were 1000 °C. Argon was employed as a diluent gas to slow down the chemical reaction rate of deposition. The test specimens were machined from fabricated composites and further coated with SiC by isothermal CVI under the same conditions. The fiber volume was *V*_f_ = 0.4, and the average tensile strength was approximately *σ*_uts_ = 225 MPa. The dog-bone shaped specimens were cut from composite panels using diamond tooling. The tension–tension fatigue tests were conducted on an MTS Model 809 servo hydraulic load-frame (MTS Systems Corp., Minneapolis, MN, USA). The fatigue experiments were performed under load control at a loading frequency *f* = 10 Hz. The fatigue load ratio (*σ*_min_/*σ*_max_) was *R* = 0.1.

**Figure 5:**
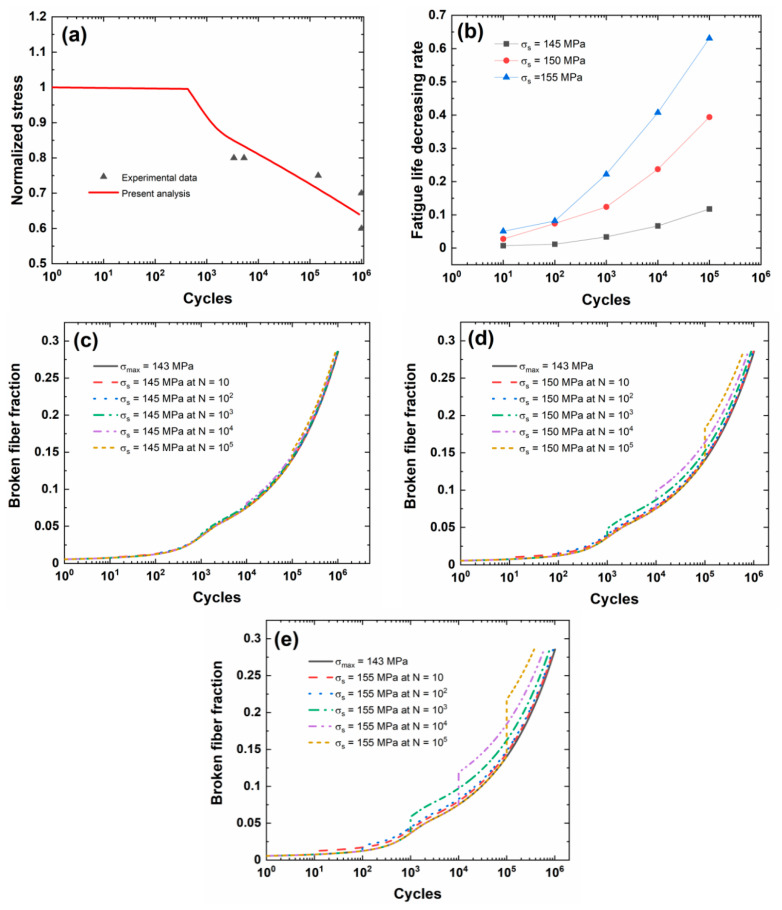
(**a**) Experimental and predicted fatigue life S−N curves; (**b**) the fatigue life decreasing rate versus occurrence applied cycle curve for different stochastic overloading stress levels; (**c**) the broken fiber fraction versus applied cycle curves under *σ*_limit_ = 143 MPa and stochastic overloading stress of *σ*_s_ = 145 MPa at *N*_s_ = 10, 10^2^, 10^3^, 10^4^, and 10^5^; (**d**) the broken fiber fraction versus applied cycle curves under *σ*_limit_ = 143 MPa and stochastic overloading stress of *σ*_s_ = 150 MPa at *N*_s_ = 10, 10^2^, 10^3^, 10^4^, and 10^5^; and, (**e**) the broken fiber fraction versus applied cycle curves under *σ*_limit_ = 143 MPa and stochastic overloading stress of *σ*_s_ = 155 MPa at *N*_s_ = 10, 10^2^, 10^3^, 10^4^, and 10^5^ of 2.5D C/SiC composite.

Figure 5a shows the experimental and predicted fatigue life S−N curves of the 2.5D C/SiC composite. When the fatigue limit applied cycle number is defined to be *N*_limit_ = 10^6^, the corresponding predicted fatigue limit stress is approximately *σ*_limit_ = 143 MPa (approximately 63.5%*σ*_uts_). The fatigue limit stress of the 2.5D C/SiC composite is lower than the other CMCs, i.e., unidirectional, cross-ply, 2D, and 3D CMCs. The low fatigue limit stress of the 2.5D C/SiC composite is mainly due to yarns bending inside of composites.

Figure 5b shows the fatigue life decreasing rate versus the occurrence applied cycle number of stochastic overloading curves for different stochastic overloading stress levels of *σ*_s_ = 145, 150, and 155 MPa (i.e., 1.014, 1.049, 1.084 fatigue limit stress). During the application of CMC components, the overloading stress level is not high, and the low overloading stress level is chosen for analysis. Under the same stochastic overloading stress level, the fatigue life decreasing rate increases with the occurrence applied cycle of stochastic overloading stress. Under *σ*_s_ = 145 MPa, the fatigue life decreasing rate increases from Λ = 0.00723 at *N*_s_ = 10 to Λ = 0.11768 at *N*_s_ = 10^5^; under *σ*_s_ = 150 MPa, the fatigue life decreasing rate increases from Λ = 0.02742 at *N*_s_ = 10 to Λ = 0.3939 at *N*_s_ = 10^5^; and, under *σ*_s_ = 155 MPa, the fatigue life decreasing rate increases from Λ = 0.05087 at *N*_s_ = 10 to Λ = 0.63095 at *N*_s_ = 10^5^. For the 2.5D C/SiC, with fatigue cycles increasing, the fatigue damage extent increases, leading to the increase in the fatigue life decreasing rate with the occurrence cycle number of stochastic overloading stress.

Figure 5c–e shows the broken fiber fraction versus applied cycle curves for different stochastic overloading stress levels and occurrence applied cycle numbers. The broken fiber fraction increases when stochastic overloading stress occurs, and the difference of the broken fiber fraction between the original peak stress and stochastic overloading stress level increases with the applied cycle.

Table 5 shows the fatigue limit stress and broken fiber fraction at different occurrence applied cycle numbers and stochastic overloading stress. When stochastic overloading stress *σ*_s_ = 145 MPa occurs at applied cycles *N*_s_ = 10, 10^2^, 10^3^, 10^4^, and 10^5^, the broken fiber fraction increases from *P*_f_ = 0.00767, 0.01251, 0.03658, 0.07519, and 0.14038 under *σ*_limit_ = 143 MPa to *P*_f_ = 0.00834, 0.01359, 0.03969, 0.08145, and 0.15161; when stochastic overloading stress *σ*_s_ = 150 MPa occurs at applied cycles *N*_s_ = 10, 10^2^, 10^3^, 10^4^, and 10^5^, the broken fiber fraction increases to *P*_f_ = 0.01021, 0.01663, 0.04842, 0.09888, and 0.1825; and, when stochastic overloading stress *σ*_s_ = 155 MPa occurs at applied cycles *N* = 10, 10^2^, 10^3^, 10^4^, and 10^5^, the broken fiber fraction increases to *P*_f_ = 0.01241, 0.02021, 0.05864, 0.11905, and 0.21754.

### 3.5. The 3D C/SiC Composite

The 3D T−300^TM^ carbon fiber-reinforced silicon carbide composite was fabricated using the chemical vapor infiltration (CVI) method. Low pressure I-CVI was employed to deposit a pyrolytic carbon layer and the silicon carbide matrix. A thin pyrolytic carbon layer was deposited on the surface of the carbon fiber as the interfacial layer with C_4_H_10_ at 950–1000 °C. The thickness of the pyrolytic carbon layer was approximately 0.2 μm. The fiber volume is *V*_f_ = 0.4, and the average tensile strength is approximately *σ*_uts_ = 276 MPa. The dog-bone shaped specimens were cut from composite panels using the diamond tooling, and then coated with a SiC coating. The tension–tension fatigue tests at room temperature were conducted on a servohydraulic mechanical testing machine. The fatigue experiments were performed under load control at a loading frequency *f* = 60 Hz. The fatigue load ratio (*σ*_min_/*σ*_max_) was *R* = 0.1. The loading frequency affects the fatigue life and fatigue limit stress. At room temperature, When the loading frequency increases, the fatigue limit stress also increases. 

**Figure 6:**
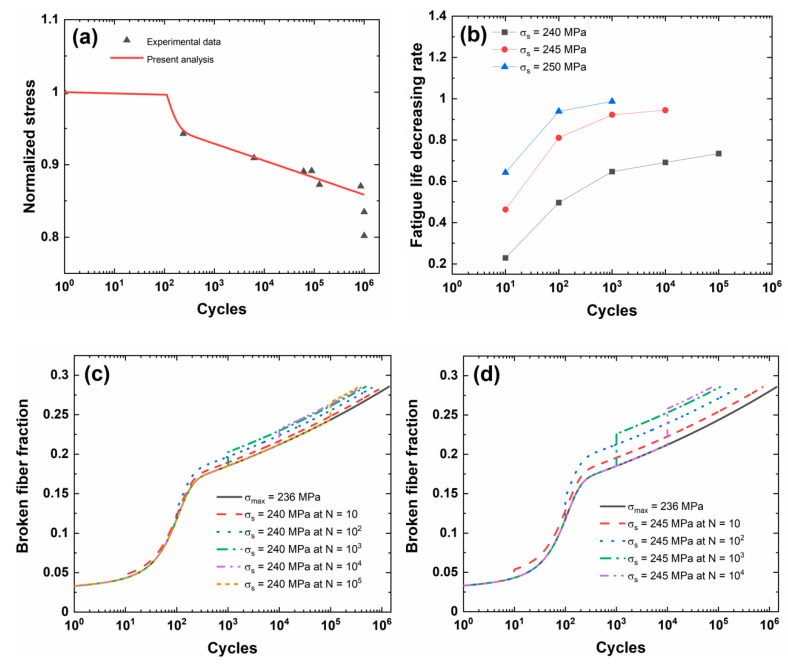

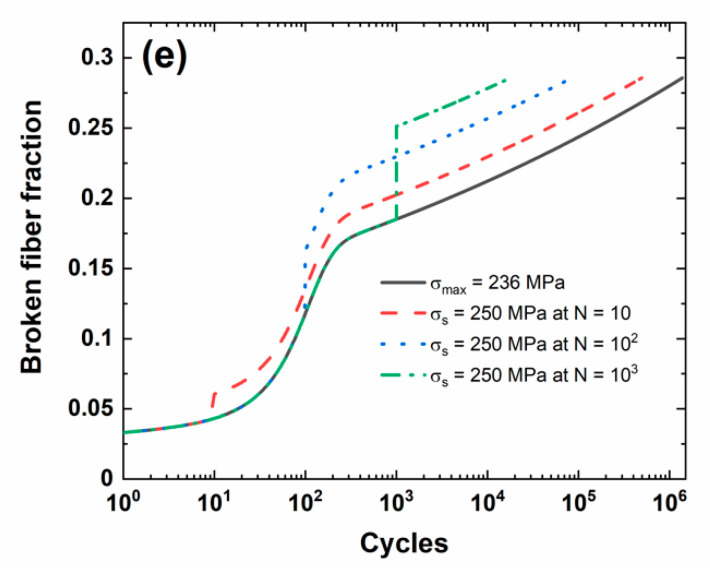
(**a**) Experimental and predicted fatigue life S−N curves; (**b**) the fatigue life decreasing rate versus occurrence applied cycle curve for different stochastic overloading stress levels; (**c**) the broken fiber fraction versus applied cycle curves under *σ*_limit_ = 236 MPa and stochastic overloading stress of *σ*_s_ = 240 MPa at *N*_s_ = 10, 10^2^, 10^3^, 10^4^, and 10^5^; (**d**) the broken fiber fraction versus applied cycle curves under *σ*_limit_ = 236 MPa and stochastic overloading stress of *σ*_s_ = 245 MPa at *N*_s_ = 10, 10^2^, 10^3^, and 10^4^; and, (**e**) the broken fiber fraction versus applied cycle curves under *σ*_limit_ = 236 MPa and stochastic overloading stress of *σ*_s_ = 250 MPa at *N*_s_ = 10, 10^2^, and 10^3^ of 3D C/SiC composite.

Figure 6a shows experimental and predicted fatigue life S−N curves of 3D C/SiC composite. When the fatigue limit applied cycle number is defined to be *N*_limit_ = 10^6^, the corresponding predicted fatigue limit stress is approximately *σ*_limit_ = 236 MPa (approximately 85.5%*σ*_uts_).

Figure 6b shows the fatigue life decreasing rate versus the occurrence cycle number of stochastic overloading curves for different stochastic overloading stress levels of *σ*_s_ = 240, 245, and 250 MPa (i.e., approximately 1.017, 1.038, and 1.059 fatigue limit stress). During application of CMC components, the overloading stress level is not high, and the low overloading stress level is chosen for analysis. Under the same stochastic overloading stress level, the fatigue life decreasing rate increases with the occurrence applied cycle of stochastic overloading. Under *σ*_s_ = 240 MPa, the fatigue life decreasing rate increases from Λ = 0.22884 at *N*_s_ = 10 to Λ = 0.73423 at *N*_s_ = 10^5^; under *σ*_s_ = 245 MPa, the fatigue life decreasing rate increases from Λ = 0.46292 at *N*_s_ = 10 to Λ = 0.94456 at *N*_s_ = 10^4^; and under *σ*_s_ = 250 MPa, the fatigue life decreasing rate increases from Λ = 0.64272 at *N*_s_ = 10 to Λ = 0.98713 at *N*_s_ = 10^3^. When the occurrence applied cycle is between *N*_s_ = 10 and 10^2^, the fatigue life decreasing rate increases rapidly; however, when the occurrence applied cycle is between *N*_s_ = 10^2^ and 10^5^, the fatigue life decreasing rate increases slowly. The occurrence of stochastic overloading stress at the initial stage of cyclic fatigue loading deteriorates the fatigue damage evolution, i.e., decreasing matrix crack spacing in transverse and longitudinal yarns, increasing interface debonding length, and broken fiber fraction; however, when matrix cracking and interface wear approach a steady-state, the effect of stochastic overloading on the fatigue damage or the fatigue life decreasing rate decreases.

Figure 6c–e shows the broken fiber fraction versus the applied cycle curves for different stochastic overloading stress levels and occurrence cycle numbers. The broken fiber fraction increases when stochastic overloading stress occurs, and the difference of the broken fiber fraction between the original peak stress and stochastic overloading stress level increases with the applied cycle.

Table 6 shows the fatigue limit stress and broken fiber fraction at different occurrence cycle numbers and stochastic overloading stress. When stochastic overloading stress *σ*_s_ = 240 MPa occurs at applied cycles *N*_s_ = 10, 10^2^, 10^3^, 10^4^, and 10^5^, the broken fiber fraction increases from *P*_f_ = 0.04324, 0.11794, 0.18509, 0.2122, and 0.24367 under *σ*_limit_ = 236 MPa to *P*_f_ = 0.04771, 0.12961, 0.2026, 0.23189, and 0.26575; when stochastic overloading stress *σ*_s_ = 245 MPa occurs at applied cycles *N*_s_ = 10, 10^2^, 10^3^, and 10^4^, the broken fiber fraction increases to *P*_f_ = 0.05383, 0.14538, 0.22602, and 0.25812; and, when stochastic overloading stress *σ*_s_ = 250 MPa occurs at applied cycles *N* = 10, 10^2^, and 10^3^, the broken fiber fraction increases to *P*_f_ = 0.06055, 0.1625, and 0.25116.

## 4. Discussion

**Figure 7:**
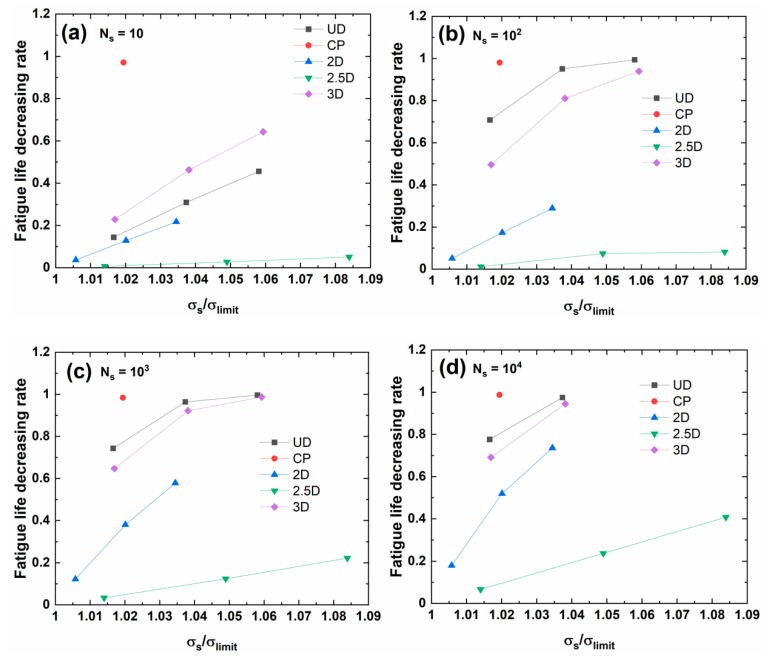

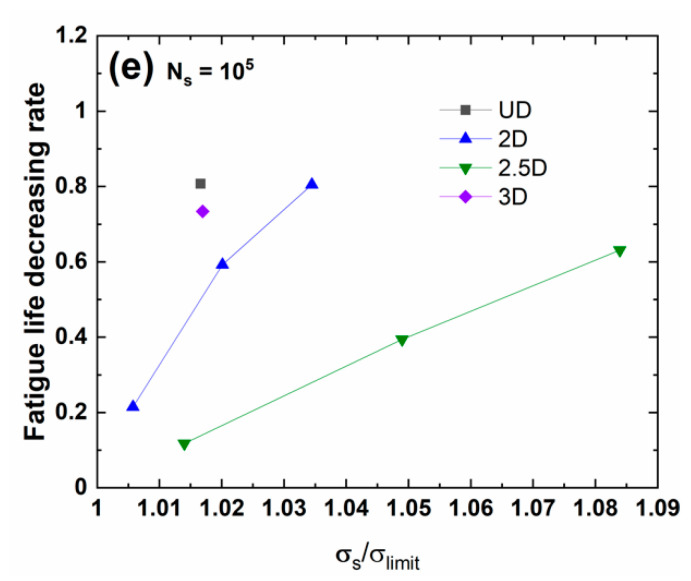
Fatigue life decreasing rate versus stochastic overloading stress for different occurrence applied cycle of (**a**) *N*_s_ = 10; (**b**) *N*_s_ = 10^2^; (**c**) *N*_s_ = 10^3^; (**d**) *N*_s_ = 10^4^; and, (**e**) *N*_s_ = 10^5^.

Figure 7 shows the fatigue life decreasing rate versus stochastic overloading stress for different occurrence cycle numbers of different C/SiC composites. The fatigue life decreasing rate increases with the stochastic overloading stress level for different fiber preforms (i.e., unidirectional, cross-ply, 2D, 2.5D, and 3D). However, with increasing applied cycles, the evolution of the fatigue life decreasing rate with stochastic overloading stress depends on the fiber preforms, which indicates that the fiber preforms affect the fatigue damage evolution process.

For *N*_s_ = 10, 10^2^, 10^3^, and 10^4^, under the same stochastic overloading stress level, the fatigue life decreasing rate is the highest for the cross-ply C/SiC composite, which indicates that the fiber preform of the cross-ply is very sensitive to the stochastic overloading stress.

For *N*_s_ = 10, 10^2^, 10^3^, 10^4^, and 10^5^, under the same stochastic overloading stress level, the fatigue life decreasing rate is the lowest for the 2.5D C/SiC composite, which indicates that the fiber preform of 2.5D has high resistance to the stochastic overloading stress.

Among UD, 2D, and 3D C/SiC composites, at the initial stage of cyclic fatigue loading, i.e., *N*_s_ = 10, under the same stochastic overloading stress, the fatigue life decrease rate of the 3D C/SiC is the highest; however, with the increasing applied cycle number, the fatigue life decreasing rate of the UD C/SiC composite is the highest under the same stochastic overloading stress.

## 5. Conclusions

In this paper, a micromechanical fatigue limit stress model of fiber-reinforced CMCs subjected to stochastic overloading stress is developed. The fatigue limit stress for different C/SiC composites is predicted. The relationships between fatigue life decreasing rate, stochastic overloading stress and corresponding occurrence cycle number, and broken fiber fraction are analyzed.

Under the same stochastic overloading stress level, the fatigue life decreasing rate increases with the stochastic overloading stress level and occurrence applied cycle of stochastic overloading for different fiber preforms. The broken fiber fraction increases when the stochastic overloading stress occurs, and the difference of the broken fiber fraction between the fatigue limit stress and stochastic overloading stress level increases with the applied cycle.

Under the same stochastic overloading stress level and occurrence applied cycle, the fatigue life decreasing rate is the highest for the cross-ply C/SiC composite, and lowest for the 2.5D C/SiC composite.

For UD, CP, and 3D C/SiC composites, when the applied cycle is between *N*_s_ = 10 and 10^2^, the fatigue life decreasing rate increases rapidly with the occurrence applied cycle; however, when the applied cycle is higher than *N*_s_ = 10^2^, the fatigue life decreasing rate increases slowly with the applied cycle.

For the 2D and 2.5D C/SiC composites, when the applied cycle is between *N*_s_ = 10 and 10^2^, the fatigue life decreasing rate increases slowly with the occurrence applied cycle; however, when the applied cycle is between *N*_s_ = 10^2^ and 10^5^, the fatigue life decreasing rate increases rapidly with the applied cycle.

## Figures and Tables

**Table 1:** Material properties of carbon fiber-reinforced silicon carbide (C/SiC) composite.

Items	Unidirectional [7]	Cross-Ply [13]	2D [10]	2.5D [8]	3D [9]
**Manufacturing Process**	Hot Pressing	Hot Pressing	Chemical Vapor Infiltration (CVI)	CVI	CVI
**Stress Ratio**	0.1	0.1	0.1	0.1	0.1
**Frequency/(Hz)**	10	10	10	10	60
**Fiber Type**	T−700^TM^	T−700^TM^	T−300^TM^	T−300^TM^	T−300^TM^
***V*_f_**	0.4	0.4	0.45	0.4	0.4
***σ*_uts_^1^/(MPa)**	270	124	420	225	276
***r*_f_^2^/(μm)**	3.5	3.5	3.5	3.5	3.5
**τ_io_^3^/(MPa)**	8	6.2	25	20	20
**τ_imin_^4^/(MPa)**	0.3	1.5	8	8	5
***ω*^5^**	0.04	0.06	0.002	0.001	0.02
**Λ ^5^**	1.5	1.8	1.0	1.0	1.0
***p*_1_^6^**	0.01	0.01	0.018	0.02	0.012
***p*_2_^6^**	1.0	0.8	1.0	1.2	1.0
***m*^7^**	5	5	5	5	5

^1^*σ*_uts_ is composite tensile strength; ^2^ *r*_f_ is the fiber radius;.^3^ τ_io_ is the interface shear stress upon initial loading; ^4^ τ_imin_ is the steady-state interface shear stress; ^5^ *ω* and *λ* are the interface degradation model parameters; ^6^ *p*_1_ and *p*_2_ are the fiber strength degradation model parameters; ^7^ *m* is the fiber Weibull modulus.

**Table 2:** Fatigue limit stress and broken fiber fraction of unidirectional C/SiC composite under stochastic overloading stress.

***σ*_max_ = 241 MPa**	***N*_f_^2^**		***N*^3^ = 1**	***N* = 10**	***N* = 10^2^**	***N* = 10^3^**	***N* = 10^4^**	***N* = 10^5^**
	**1,431,993**	***P*_f_**	**0.00609**	**0.02113**	**0.17991**	**0.20077**	**0.22431**	**0.25085**
*σ*_s_^1^ = 245 MPa *N* = 10	*N*_f_		*N* = 1	*N* = 10	*N* = 10^2^	*N* = 10^3^	*N* = 10^4^	*N* = 10^5^
	1,225,895	*P*_f_	0.00609	0.02329	0.18207	0.20294	0.22648	0.25302
*σ*_s_ = 245 MPa *N* = 10^2^	*N*_f_		*N* = 1	*N* = 10	*N* = 10^2^	*N* = 10^3^	*N* = 10^4^	*N* = 10^5^
	418,087	*P*_f_	0.00609	0.02113	0.19662	0.21749	0.24103	0.26757
*σ*_s_ = 245 MPa *N* = 10^3^	*N*_f_		*N* = 1	*N* = 10	*N* = 10^2^	*N* = 10^3^	*N* = 10^4^	*N* = 10^5^
	368,186	*P*_f_	0.00609	0.02113	0.17991	0.21915	0.24269	0.26923
*σ*_s_ = 245 MPa *N* = 10^4^	*N*_f_		*N* = 1	*N* = 10	*N* = 10^2^	*N* = 10^3^	*N* = 10^4^	*N* = 10^5^
	320,486	*P*_f_	0.00609	0.02113	0.17991	0.20077	0.2445	0.27104
*σ*_s_ = 245 MPa *N* = 10^5^	*N*_f_		*N* = 1	*N* = 10	*N* = 10^2^	*N* = 10^3^	*N* = 10^4^	*N* = 10^5^
	275,798	*P*_f_	0.00609	0.02113	0.17991	0.20077	0.22431	0.27298
*σ*_s_ = 250 MPa *N* = 10	*N*_f_		*N* = 1	*N* = 10	*N* = 10^2^	*N* = 10^3^	*N* = 10^4^	*N* = 10^5^
	989,365	*P*_f_	0.00609	0.02626	0.18504	0.2059	0.22944	0.25598
*σ*_s_ = 250 MPa *N* = 10^2^	*N*_f_		*N* = 1	*N* = 10	*N* = 10^2^	*N* = 10^3^	*N* = 10^4^	*N* = 10^5^
	70,700	*P*_f_	0.00609	0.02113	0.21897	0.23983	0.26338	0.28571
*σ*_s_ = 250 MPa *N* = 10^3^	*N*_f_		*N* = 1	*N* = 10	*N* = 10^2^	*N* = 10^3^	*N* = 10^4^	*N* = 10^5^
	51,311	*P*_f_	0.00609	0.02113	0.17991	0.24365	0.26719	0.28571
*σ*_s_ = 250 MPa *N* = 10^4^	*N*_f_		*N* = 1	*N* = 10	*N* = 10^2^	*N* = 10^3^	*N* = 10^4^	*N* = 10^5^
	36,095	*P*_f_	0.00609	0.02113	0.17991	0.20077	0.27131	0.28571
*σ*_s_ = 255 MPa *N* = 10	*N*_f_		*N* = 1	*N* = 10	*N* = 10^2^	*N* = 10^3^	*N* = 10^4^	*N* = 10^5^
	779,144	*P*_f_	0.00609	0.02952	0.1883	0.20917	0.23271	0.25925
*σ*_s_ = 255 MPa *N* = 10^2^	*N*_f_		*N* = 1	*N* = 10	*N* = 10^2^	*N* = 10^3^	*N* = 8593	
	8593	*P*_f_	0.00609	0.02113	0.24295	0.26381	0.28571	
*σ*_s_ = 255 MPa *N* = 10^3^	*N*_f_		*N* = 1	*N* = 10	*N* = 10^2^	*N* = 10^3^	*N* = 4870	
	4870	*P*_f_	0.00609	0.02113	0.17991	0.26984	0.28571	

^1^*σ*_s_ is stochastic overloading stress; ^2^ *N*_f_ is the cycle number corresponding to fatigue fracture; ^3^ *N* is applied cycle.

**Table 3:** Fatigue limit stress and broken fiber fraction of cross-ply C/SiC composite under stochastic overloading stress.

σ_max_ = 103 MPa	Nf		N = 1	N = 10	N = 102	N = 103	N = 104	N = 105
	1,568,296	*P*_f_	0.05349	0.20047	0.22748	0.24133	0.25508	0.26892
*σ*_s_ = 105 MPa *N* = 10	*N*_f_		*N* = 1	*N* = 10	*N* = 10^2^	*N* = 10^3^	*N* = 10^4^	*N* = 45,256
	45,256	*P*_f_	0.05349	0.22205	0.24906	0.26291	0.27666	0.28571
*σ*_s_ = 105 MPa *N* = 10^2^	*N*_f_		*N* = 1	*N* = 10	*N* = 10^2^	*N* = 10^3^	*N* = 10^4^	*N* = 30,243
	30,243	*P*_f_	0.05349	0.20047	0.25148	0.26533	0.27908	0.28571
*σ*_s_ = 105 MPa *N* = 10^3^	*N*_f_		*N* = 1	*N* = 10	*N* = 10^2^	*N* = 10^3^	*N* = 10^4^	*N* = 24,787
	24,787	*P*_f_	0.05349	0.20047	0.22748	0.26653	0.28028	0.28571
*σ*_s_ = 105 MPa *N* = 10^4^	*N*_f_		*N* = 1	*N* = 10	*N* = 10^2^	*N* = 10^3^	*N* = 10^4^	*N* = 20,455
	20,455	*P*_f_	0.05349	0.20047	0.22748	0.24133	0.28143	0.28571

**Table 4:** Fatigue limit stress and broken fiber fraction of 2D C/SiC composite under stochastic overloading stress.

*σ*_max_ = 348 MPa	*N*_f_		*N* = 1	*N* = 10	*N* = 10^2^	*N* = 10^3^	*N* = 10^4^	*N* = 10^5^
	1,067,612	*P*_f_	0.02529	0.03153	0.04397	0.11358	0.1795	0.22527
*σ*_s_ = 350 MPa *N* = 10	*N*_f_		*N* = 1	*N* = 10	*N* = 10^2^	*N* = 10^3^	*N* = 10^4^	*N* = 10^5^
	1,028,121	*P*_f_	0.02529	0.03261	0.04505	0.11466	0.18059	0.22635
*σ*_s_ = 350 MPa *N* = 10^2^	*N*_f_		*N* = 1	*N* = 10	*N* = 10^2^	*N* = 10^3^	*N* = 10^4^	*N* = 10^5^
	1,013,262	*P*_f_	0.02529	0.03153	0.04547	0.11508	0.18101	0.22677
*σ*_s_ = 350 MPa *N* = 10^3^	*N*_f_		*N* = 1	*N* = 10	*N* = 10^2^	*N* = 10^3^	*N* = 10^4^	*N* = 10^5^
	937,226	*P*_f_	0.02529	0.03153	0.04397	0.11731	0.18323	0.229
*σ*_s_ = 350 MPa *N* = 10^4^	*N*_f_		*N* = 1	*N* = 10	*N* = 10^2^	*N* = 10^3^	*N* = 10^4^	*N* = 10^5^
	875,603	*P*_f_	0.02529	0.03153	0.04397	0.11358	0.18516	0.23093
*σ*_s_ = 350 MPa *N* = 10^5^	*N*_f_		*N* = 1	*N* = 10	*N* = 10^2^	*N* = 10^3^	*N* = 10^4^	*N* = 10^5^
	838,274	*P*_f_	0.02529	0.03153	0.04397	0.11358	0.1795	0.23215
*σ*_s_ = 355 MPa *N* = 10	*N*_f_		*N* = 1	*N* = 10	*N* = 10^2^	*N* = 10^3^	*N* = 10^4^	*N* = 10^5^
	930,683	*P*_f_	0.02529	0.03546	0.0479	0.11751	0.18342	0.2292
*σ*_s_ = 355 MPa *N* = 10^2^	*N*_f_		*N* = 1	*N* = 10	*N* = 10^2^	*N* = 10^3^	*N* = 10^4^	*N* = 10^5^
	882,390	*P*_f_	0.02529	0.03153	0.04941	0.11902	0.18493	0.23071
*σ*_s_ = 355 MPa *N* = 10^3^	*N*_f_		*N* = 1	*N* = 10	*N* = 10^2^	*N* = 10^3^	*N* = 10^4^	*N* = 10^5^
	661,586	*P*_f_	0.02529	0.03153	0.04397	0.12704	0.19295	0.23873
*σ*_s_ = 355 MPa *N* = 10^4^	*N*_f_		*N* = 1	*N* = 10	*N* = 10^2^	*N* = 10^3^	*N* = 10^4^	*N* = 10^5^
	513,025	*P*_f_	0.02529	0.03153	0.04397	0.11358	0.19983	0.24561
*σ*_s_ = 355 MPa *N* = 10^5^	*N*_f_		*N* = 1	*N* = 10	*N* = 10^2^	*N* = 10^3^	*N* = 10^4^	*N* = 10^5^
	435,308	*P*_f_	0.02529	0.03153	0.04397	0.11358	0.17949	0.24996
*σ*_s_ = 360 MPa *N* = 10	*N*_f_		*N* = 1	*N* = 10	*N* = 10^2^	*N* = 10^3^	*N* = 10^4^	*N* = 10^5^
	835,692	*P*_f_	0.02529	0.0385	0.05094	0.12055	0.18646	0.23224
*σ*_s_ = 360 MPa *N* = 10^2^	*N*_f_		*N* = 1	*N* = 10	*N* = 10^2^	*N* = 10^3^	*N* = 10^4^	*N* = 10^5^
	759,497	*P*_f_	0.02529	0.03153	0.05361	0.12323	0.18914	0.23491
*σ*_s_ = 360 MPa *N* = 10^3^	*N*_f_		*N* = 1	*N* = 10	*N* = 10^2^	*N* = 10^3^	*N* = 10^4^	*N* = 10^5^
	450,695	*P*_f_	0.02529	0.03153	0.04397	0.13736	0.20327	0.24905
*σ*_s_ = 360 MPa *N* = 10^4^	*N*_f_		*N* = 1	*N* = 10	*N* = 10^2^	*N* = 10^3^	*N* = 10^4^	*N* = 10^5^
	282,293	*P*_f_	0.02529	0.03153	0.04397	0.11358	0.2153	0.26108
*σ*_s_ = 360 MPa *N* = 10^5^	*N*_f_		*N* = 1	*N* = 10	*N* = 10^2^	*N* = 10^3^	*N* = 10^4^	*N* = 10^5^
	208,007	*P*_f_	0.02529	0.03153	0.04397	0.11358	0.17949	0.26861

**Table 5:** Fatigue limit stress and broken fiber fraction of 2.5D C/SiC composite under stochastic overloading stress.

*σ*_max_ = 143 MPa	*N*_f_		*N* = 1	*N* = 10	*N* = 10^2^	*N* = 10^3^	*N* = 10^4^	*N* = 10^5^
	1,012,346	*P*_f_	0.00574	0.00767	0.01251	0.03658	0.07519	0.14038
*σ*_s_ = 145 MPa *N* = 10	*N*_f_		*N* = 1	*N* = 10	*N* = 10^2^	*N* = 10^3^	*N* = 10^4^	*N* = 10^5^
	1,005,026	*P*_f_	0.00574	0.00834	0.01317	0.03724	0.07585	0.14105
*σ*_s_ = 145 MPa *N* = 10^2^	*N*_f_		*N* = 1	*N* = 10	*N* = 10^2^	*N* = 10^3^	*N* = 10^4^	*N* = 10^5^
	1,000,463	*P*_f_	0.00574	0.00767	0.01359	0.03766	0.07627	0.14146
*σ*_s_ = 145 MPa *N* = 10^3^	*N*_f_		*N* = 1	*N* = 10	*N* = 10^2^	*N* = 10^3^	*N* = 10^4^	*N* = 10^5^
	978,311	*P*_f_	0.00574	0.00767	0.01251	0.03969	0.0783	0.1435
*σ*_s_ = 145 MPa *N* = 10^4^	*N*_f_		*N* = 1	*N* = 10	*N* = 10^2^	*N* = 10^3^	*N* = 10^4^	*N* = 10^5^
	944,705	*P*_f_	0.00574	0.00767	0.01251	0.03658	0.08145	0.14664
*σ*_s_ = 145 MPa *N* = 10^5^	*N*_f_		*N* = 1	*N* = 10	*N* = 10^2^	*N* = 10^3^	*N* = 10^4^	*N* = 10^5^
	893,212	*P*_f_	0.00574	0.00767	0.01251	0.03658	0.07519	0.15161
*σ*_s_ = 150 MPa *N* = 10	*N*_f_		*N* = 1	*N* = 10	*N* = 10^2^	*N* = 10^3^	*N* = 10^4^	*N* = 10^5^
	984,589	*P*_f_	0.00574	0.01021	0.01504	0.03911	0.07772	0.14292
*σ*_s_ = 150 MPa *N* = 10^2^	*N*_f_		*N* = 1	*N* = 10	*N* = 10^2^	*N* = 10^3^	*N* = 10^4^	*N* = 10^5^
	967,500	*P*_f_	0.00574	0.00767	0.01663	0.0407	0.07931	0.1445
*σ*_s_ = 150 MPa *N* = 10^3^	*N*_f_		*N* = 1	*N* = 10	*N* = 10^2^	*N* = 10^3^	*N* = 10^4^	*N* = 10^5^
	886,901	*P*_f_	0.00574	0.00767	0.01251	0.04842	0.08703	0.15223
*σ*_s_ = 150 MPa *N* = 10^4^	*N*_f_		*N* = 1	*N* = 10	*N* = 10^2^	*N* = 10^3^	*N* = 10^4^	*N* = 10^5^
	772,084	*P*_f_	0.00574	0.00767	0.01251	0.03658	0.09888	0.16408
*σ*_s_ = 150 MPa *N* = 10^5^	*N*_f_		*N* = 1	*N* = 10	*N* = 10^2^	*N* = 10^3^	*N* = 10^4^	*N* = 10^5^
	613,579	*P*_f_	0.00574	0.00767	0.01251	0.03658	0.07519	0.1825
*σ*_s_ = 155 MPa *N* = 10	*N*_f_		*N* = 1	*N* = 10	*N* = 10^2^	*N* = 10^3^	*N* = 10^4^	*N* = 10^5^
	960,848	*P*_f_	0.00574	0.01241	0.01725	0.04132	0.07993	0.14512
*σ*_s_ = 155 MPa *N* = 10^2^	*N*_f_		*N* = 1	*N* = 10	*N* = 10^2^	*N* = 10^3^	*N* = 10^4^	*N* = 10^5^
	929,613	*P*_f_	0.00574	0.00767	0.02021	0.04427	0.08288	0.14808
*σ*_s_ = 155 MPa *N* = 10^3^	*N*_f_		*N* = 1	*N* = 10	*N* = 10^2^	*N* = 10^3^	*N* = 10^4^	*N* = 10^5^
	787,301	*P*_f_	0.00574	0.00767	0.01251	0.05864	0.09725	0.16245
*σ*_s_ = 155 MPa *N* = 10^4^	*N*_f_		*N* = 1	*N* = 10	*N* = 10^2^	*N* = 10^3^	*N* = 10^4^	*N* = 10^5^
	599,723	*P*_f_	0.00574	0.00767	0.01251	0.03658	0.11905	0.18425
*σ*_s_ = 155 MPa *N* = 10^5^	*N*_f_		*N* = 1	*N* = 10	*N* = 10^2^	*N* = 10^3^	*N* = 10^4^	*N* = 10^5^
	373,608	*P*_f_	0.00574	0.00767	0.01251	0.03658	0.07519	0.21754

**Table 6:** Fatigue limit stress and broken fiber fraction of 3D C/SiC composite under stochastic overloading stress.

*σ*_max_ = 236 MPa	*N*_f_		*N* = 1	*N* = 10	*N* = 10^2^	*N* = 10^3^	*N* = 10^4^	*N* = 10^5^
	1,383,192	*P*_f_	0.03314	0.04324	0.11794	0.18509	0.2122	0.24367
*σ*_s_ = 240 MPa *N* = 10	*N*_f_		*N* = 1	*N* = 10	*N* = 10^2^	*N* = 10^3^	*N* = 10^4^	*N* = 10^5^
	1,066,666	*P*_f_	0.03314	0.04771	0.12242	0.18957	0.21668	0.24815
*σ*_s_ = 240 MPa *N* = 10^2^	*N*_f_		*N* = 1	*N* = 10	*N* = 10^2^	*N* = 10^3^	*N* = 10^4^	*N* = 10^5^
	696,321	*P*_f_	0.03314	0.04324	0.12961	0.19676	0.22387	0.25534
*σ*_s_ = 240 MPa *N* = 10^3^	*N*_f_		*N* = 1	*N* = 10	*N* = 10^2^	*N* = 10^3^	*N* = 10^4^	*N* = 10^5^
	488,262	*P*_f_	0.03314	0.04324	0.11794	0.2026	0.22971	0.26118
*σ*_s_ = 240 MPa *N* = 10^4^	*N*_f_		*N* = 1	*N* = 10	*N* = 10^2^	*N* = 10^3^	*N* = 10^4^	*N* = 10^5^
	426,789	*P*_f_	0.03314	0.04324	0.11794	0.18509	0.23189	0.26336
*σ*_s_ = 240 MPa *N* = 10^5^	*N*_f_		*N* = 1	*N* = 10	*N* = 10^2^	*N* = 10^3^	*N* = 10^4^	*N* = 10^5^
	367,609	*P*_f_	0.03314	0.04324	0.11794	0.18509	0.2122	0.26575
*σ*_s_ = 245 MPa *N* = 10	*N*_f_		*N* = 1	*N* = 10	*N* = 10^2^	*N* = 10^3^	*N* = 10^4^	*N* = 10^5^
	742,891	*P*_f_	0.03314	0.05383	0.12853	0.19568	0.22279	0.25426
*σ*_s_ = 245 MPa *N* = 10^2^	*N*_f_		*N* = 1	*N* = 10	*N* = 10^2^	*N* = 10^3^	*N* = 10^4^	*N* = 10^5^
	262,035	*P*_f_	0.03314	0.04324	0.14538	0.21253	0.23963	0.2711
*σ*_s_ = 245 MPa *N* = 10^3^	*N*_f_		*N* = 1	*N* = 10	*N* = 10^2^	*N* = 10^3^	*N* = 10^4^	*N* = 10^5^
	107,854	*P*_f_	0.03314	0.04324	0.11794	0.22602	0.25313	0.2846
*σ*_s_ = 245 MPa *N* = 10^4^	*N*_f_		*N* = 1	*N* = 10	*N* = 10^2^	*N* = 10^3^	*N* = 10^4^	*N* = 10^5^
	76,689	*P*_f_	0.03314	0.04324	0.11794	0.18509	0.25812	0.28571
*σ*_s_ = 250 MPa *N* = 10	*N*_f_		*N* = 1	*N* = 10	*N* = 10^2^	*N* = 10^3^	*N* = 10^4^	*N* = 10^5^
	494,186	*P*_f_	0.03314	0.06055	0.13525	0.2024	0.22951	0.26098
*σ*_s_ = 250 MPa *N* = 10^2^	*N*_f_		*N* = 1	*N* = 10	*N* = 10^2^	*N* = 10^3^	*N* = 10^4^	*N* = 10^5^
	84,191	*P*_f_	0.03314	0.04324	0.1625	0.22966	0.25676	0.28571
*σ*_s_ = 250 MPa *N* = 10^3^	*N*_f_		*N* = 1	*N* = 10	*N* = 10^2^	*N* = 10^3^	*N* = 10^4^	*N* = 10^5^
	17,798	*P*_f_	0.03314	0.04324	0.11794	0.25116	0.27827	0.28571
